# Prevention of COVID-19 transmission from deceased subject: A critical point of view

**DOI:** 10.7189/jogh.12.03037

**Published:** 2022-07-16

**Authors:** Federica Mele, Valeria Santoro, Sara Sablone, Diana Logrado, Caterina Berterame, Mariagrazia Calvano, Mirko Leonardelli, Enrica Macorano, Stefano Duma, Marina G Introna, Francesco Introna, Antonio De Donno

**Affiliations:** 1 Department of Interdisciplinary Medicine, Section of Legal Medicine, Policlinico di Bari Hospital, University of Bari, Bari, Italy; 2 National Institute of Legal Medicine and Forensic Sciences, South Branch, Lisbon, Portugal

On December 31, 2019, China reported to the WHO cases of pneumonia in Wuhan caused by a novel coronavirus (2019-nCoV). This escalated into an unprecedented outbreak of infections, leading the WHO to characterise it as a pandemic on March 11, 2020. The COVID-19 pandemic is rapidly evolving, and since October 9, 2021, almost 200 000 000 people have been infected with more than 4 000 000 deaths globally [1]. To reduce transmission and mortality, efforts have been made to develop vaccines against COVID-19. Since December 2020, new vaccines have been authorized to help control the spread of the virus [2]. Despite the vaccination campaign progressing successfully, our battle is not yet won, and avoiding exposure to this virus is crucial to preventing COVID-19. Person-to-person transmission occurs primarily via direct contact or through droplets spread by the infected individual coughing or sneezing [3-5]. Although the infection rate from dead bodies is unclear [4], managing them is of great importance in reducing viral transmission, especially because mortuary staff and pathologists will be exposed to potentially infective materials from cadavers, transmitted through airways, body bags, autopsy tables, and autopsy room walls [4,6]. For this reason, many societies, institutes, and governments have given general infection and prevention advice.

This viewpoint aims to summarize how different countries manage the COVID-19 dead and perform autopsies and whether common prevention recommendations can be identified.

The PubMed database was searched until October 9, 2021. We used keywords for: deceased covid 19, management of death covid 19, autopsy covid 19 ((deceased covid 19) AND management of death covid 19) AND autopsy covid 19). The selected articles’ references were also screened. Only documents in English were included, while reviews were excluded.

## FUNERAL SERVICES

For the management of bodies, WHO recommends that health care workers or mortuary staff wear appropriate personal protective equipment (PPE) including gloves, impermeable disposable gowns, medical masks, and eye protection; that the deceased’s family can only view the body without physical contact, always using standard precautions. Furthermore, WHO does not recommend embalming to avoid excessive manipulation of the body [7].

**Figure:**
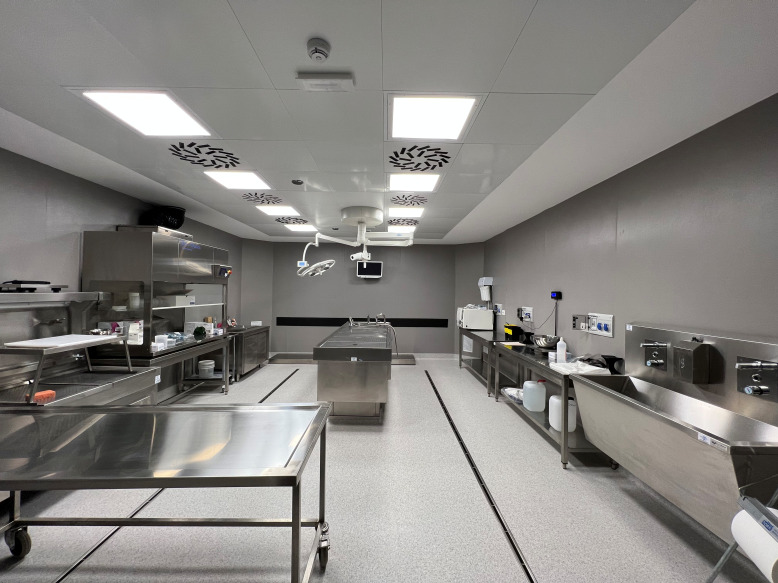
Photo: BSL3 autopsy facility. From the first author’s personal collection. Used with permission.

CDC recommends that people not touch the body of a person who died of COVID-19, especially those of older people and people with severe underlying health conditions; wearing at minimum disposable gloves is also recommended. They also recommended embalming bodies and following the standard precautions with additional PPE if splashing is expected (disposable gown and face shield or goggles and facemask) [8].

ECDC states that unnecessary contact with bodies should be minimized and that persons in direct contact with those deceased of COVID-19 should be protected by wearing appropriate PPE: gloves and a long-sleeved water-resistant gown [9]. ECDC recommendations also include administrative measures for transportation and the storage of bodies and for postmortem care such as the use of appropriate standard precautions and PPE (gloves and long-sleeved water-resistant gown), transport, and preparation of the body. Furthermore, ECDC allows for contact with the bodies and embalming of the cadavers.

In Europe, recommendations for funeral services were released from the UK, Ireland, and Italy. The UK Government, quite in contrast with the above-mentioned recommendations, stated that residual hazards from body fluid spillage do not present a risk. It also recommended the use of disposable gloves, disposable plastic aprons, surgical masks, and disposable eye protection [10].

In Ireland, the Coroner Service [11] recommends the 2013 Guidelines from the Health Service Executive and the Health Protection Surveillance Centre for Handling of Remains, as COVID-19 is currently classified as a biological agent (Hazard Group 3). The Ireland Guidelines stress the great importance of applying standard precautions (handwashing, no smoking or eating, use of appropriate protective clothing such as waterproof aprons, gowns, gloves, overshoes or Wellington boots, and appropriate eye protection) and encouraged embalming procedures.

In Italy, Fineschi et al. [12] published an operating procedure, more restrictive than the above-mentioned ones: they suggest using a sanitised metal stretcher and sheets soaked in disinfectant solution. Furthermore, they recommend the suspension of any ceremonial rites.

Restrictive recommendations were also adopted by other countries in other parts of the world. The National Institute of Forensic Medicine in Malaysia [13] states that relatives are strictly forbidden from touching, kissing, or handling the body under any circumstances. They also suggest wrapping the body with white cotton linen, placing it in a body bag, and wiping it with 0.5% sodium hypochlorite/disinfectant. Attendants must wear N95/N100 masks, gloves, and protective aprons. Disposing of the bodies through burial or cremation as soon as practicable is recommended; embalming must be avoided, and relatives are prohibited from opening the sealed coffin [13].

Restrictive guidelines were also adopted by the Government of India [14]: the deceased’s family could view the body only with the application of standard precautions; the dead body could be placed in leak-proof plastic body bag, decontaminating the exterior with 1% hypochlorite and wrapping the body-bag with a mortuary sheet, embalming should not be allowed.

The Washington State Department of Health assessed that mortuary and funeral home workers should use Standard Precautions: nonsterile, nitrile gloves and heavy-duty gloves over the nitrile ones if there is a risk of injuries that break the skin; clean, long-sleeved fluid-resistant or impermeable gown to protect skin and clothing; plastic face shield or a face mask and goggles. [15]

## AUTOPSIES

These recommendations are summarized in **Table 1****.**

**Table 1:** Summary of the recommendations about the autopsies.

	WHO	CDC	RoyC	UK	Ire	ISS	Ita	Ind	Mal
**Hazard group**	NA	NA	HG3	NA	NA	NA	HG3	NA	NA
**Autopsy room**	Ventilation	AIIR	Ventilation	NA	NA	BSL3	Ventilation	Negative pressure	BSL2
**Post-mortem swabs**	NA	Yes	Yes	Yes	NA	Yes	Yes	NA	Yes
**Tissues**	NA		NA	NA	NA			NA	
Lungs		Yes				Yes	Yes		Yes
Upper airway		Yes				Yes	Yes		NA
Liver		Yes				Yes	Opt		NA
Spleen		Yes				Yes	NA		NA
Kidney		Yes				Yes	NA		NA
Heart		Yes				Yes	NA		NA
GI tract		Yes				Yes	NA		NA
Muscles		NA				Yes	Opt		NA
Brain		NA				Yes	NA		NA
Lymph-nodes		NA				Yes	NA		NA
Pancreas		NA				Yes	NA		NA
Oscillating saw	NA	Vacuum	Vacuum	NA	NA	NA	Vacuum	Vacuum	NA
**Autopsy**	NA	NA	NA	NA					Yes
Ante-mortem confirmed diagnosis					Not required	Limited	Discretionary	Avoid	
COVID-19 neg					Autopsy				
Diagnosis not confirmed							Mandatory		
**PPE for pathologist**									
Scrub suit	Yes	Yes	Yes	NA	NA	Yes	NA	NA	Yes
Gown	Yes	Yes	Yes	Yes	NA	Yes	Yes	Yes	Yes
Gloves	3 pairs	3 pairs	2 pairs	Yes	NA	3 pairs	3 pairs	NA	3 pairs
Eye protection	Yes	Yes	Yes	Yes	NA	Yes	Yes	Yes	NA
Overshoes/boots	Yes	Yes	Yes	NA	NA	Yes	Yes	Yes	Yes
Mask	Yes	Yes	Yes	Yes		Yes	Yes	Yes	Yes
Apron	NA	Yes	Yes	Yes		Yes	NA	NA	Yes
Surgical cap	NA	Yes	Yes	NA		Option	Yes	Yes	NA

WHO – World Health Organization [7], CDC – Centre of Disease Control [16], RoyC – Royal College of Pathologist [17], UK – UK Government [10], Ire – Coroner Service of Ireland [11], ISS – Italian National Institute of Health [18], Ita-Fineschi et al [12], Ind – Indian guidelines [14], Mal – National Institute of Forensic Medicine in Malaysia [13], Yes – recommended, NA – not assessed, Opt – optional, GI tract – gastrointestinal tract, HG3 – hazard group 3, BSL – biosafety level

WHO recommendations [7] contain procedures used for any autopsies of people who have died from an acute respiratory illness: additional respiratory protection is needed such as an adequately ventilated room and appropriate PPE (scrub suit, long-sleeved fluid-resistant gown, gloves – either two pairs or one pair autopsy gloves, face shield or goggles, boots, and N95 mask).

CDC released recommendations [16] focusing on specimen collection: post-mortem swab for COVID-19 testing (nasopharyngeal and lung swab), swab for other respiratory pathogens testing, other post-mortem testing, and formalin-fixed tissue autopsy. The suggested specimens should consist of a minimum of eight blocks and fixed tissue specimens from respiratory sites (trachea, proximal and distal; hilar lung with segmental bronchi, right and left primary bronchi; representative pulmonary parenchyma from the right and left lung) and from major organs (including liver, spleen, kidney, heart, gastrointestinal tract) and any other tissues showing significant gross pathology. The CDC also suggest the avoidance of aerosol-generating procedures, such as the use of an oscillating bone saw, which should be replaced by hand shears as an alternative cutting tool. CDC recommended Airborne Infection Isolation Rooms (AIIRs) and the use of double surgical gloves interposed with a layer of cut-proof synthetic mesh gloves, fluid-resistant or impermeable gowns, waterproof aprons, goggles or face shields, N-95 respirators or higher, surgical scrubs, shoe covers, and surgical caps.

The Royal College of Pathologists’ recommendations [17] indicate that having a separate high-risk suite is ideal but not mandatory and that good ventilation is required. PPE equipment should include a surgical scrub suit, hat, clear visor, respiratory protection, a waterproof gown covering the entire body, a plastic apron over the waterproof gown, boots, gloves, and protective cut-resistant gloves. These recommendations also suggest the utility of limited autopsies (ie, needle sampling or single opening organ sampling). Samples for cases of COVID-19 consist of upper respiratory tract swabs (nose and throat swabs), lower respiratory tract swabs (sputum, BAL or lung), and blood for serology. Furthermore, they recommend taking a full set of tissue samples for histology.

The UK Government guidance [10] requires PPE consisting of disposable gloves, a disposable plastic apron, a disposable gown, a FFP3 respirator, and disposable eye protection. They recommend collecting upper respiratory tract samples (throat and nose or a nasopharyngeal aspirate) and lower respiratory tract samples (sputum) if obtainable.

The Irish Coroner Service [11] suggests that in case of confirmed antemortem diagnosis of COVID-19, a post-mortem examination will not be required. In case of patient death from respiratory failure/adult respiratory distress syndrome, post-mortem viral swabs may be taken. If negative, it is possible to proceed to a post-mortem examination.

The Italian National Institute of Health [18] suggest limiting as much as possible the performing of autopsies in subjects suspected of having contracted SARS-CoV-2 infection. Autopsies must be performed in facilities that guarantee safety standards (BSL3). They suggest that pathologists and technicians must wear scrub suits, disposable liquid-resistant gowns, disposable plastic aprons, eye protection, facial masks, surgical caps (optional), gloves (three pairs: external and internal latex gloves and in the middle a pair of cut-resistant gloves), and boots. Collecting nasopharyngeal and oropharyngeal swabs and a swab from each lung for SARS-CoV-2 testing is recommended. For histological analysis, they recommended collecting heart, lungs (parenchyma and bronchi), brain, kidneys, liver, peribronchial lymph nodes, intestinal mucosa, spleen, pancreas, and muscle samples. In Italy, Fineschi et al. [12] released other recommendations: they recommend that PPE for autopsy investigation should include disposable headgear, disposable gloves (double pair), cut-resistant protective gloves, respiratory filter FFP3, goggles or protective visor, disposable long-sleeved gowns or waterproof suits, and disposable overshoes. They recommend preliminary nasopharyngeal and oropharyngeal swabs for suspected infection. If positive, the execution of the autopsy investigation is discretionary and if the swab is negative, the autopsy is mandatory. The discretionary autopsy depends on the need to avoid contamination. They suggest performing an autopsy following *en bloc* organ extraction to adequately define the respiratory pathological manifestations. They recommended the use of an oscillating saw with bone aerosol aspiration and effective ventilation in the autopsy room. They also recommended the collection of upper respiratory tract swabs (nasopharyngeal and oropharyngeal) and lower respiratory tract swabs (from each lung) and that organ and tissue samples be kept in formalin for subsequent histopathological investigations.

Indian recommendations [14] firmly assess those autopsies should be avoided. If an autopsy is to be performed, they suggest the use of a full complement of PPE (coveralls, head cover, shoe cover, mask, goggles/face shield), that only one body cavity at a time be dissected, and that negative pressure is maintained in the mortuary.

Malaysian interim guidelines [13] recommend wearing PPE while handling the body: disposable scrub suit, disposable waterproof coverall/jump-suit with full feet cover, knee length boots, disposable shoe covers, disposable plastic apron, cut-resistant gloves, double gloves, and full-face Powered Air Purifying Respirators (PAPR) with HEPA filters. They recommend the collection of following specimens: lower respiratory tract specimens (deep cough sputum, bronchoalveolar lavage, tracheal aspirate, pleural fluid, lung tissue), upper respiratory tract specimens (nasopharyngeal and oropharyngeal swabs), and serum specimens.

## CONCLUSION

COVID-19 is the most important health challenge today and the adherence to control strategies to contain the spread of the virus is a global priority. Even if there is no consensus on the spread of the virus from the deceased person, the careful management of the bodies is mandatory, considering that the rate of contamination of personal protective equipment after full autopsies varies between 15% and 65% [4,5]. Nevertheless, this viewpoint underlines that there is no consensus on the practices and techniques for the autopsy and for the management of the body for the funeral services. Almost all the recommendations found are in contrast with each other regarding almost all the practices or their universal applicability. Even if other studies tried to indicate the minimum level of PPE and autopsy room equipment necessary for COVID-19 autopsy, the development of guidelines shared by as many scientific societies as possible is desirable.
